# Editorial: Cell-mediated immunity in ruminants: Novel approaches and insights

**DOI:** 10.3389/fvets.2023.1177315

**Published:** 2023-03-24

**Authors:** Rodrigo Prado Martins, Fernando Nogueira Souza

**Affiliations:** ^1^ISP, INRAE, Université de Tours, UMR1282, Nouzilly, France; ^2^Veterinary Clinical Immunology Research Group, Departamento de Clínica Médica, Faculdade de Medicina Veterinária e Zootecnia, Universidade de São Paulo, São Paulo, Brazil

**Keywords:** adaptive immunity, immunoassays, antigen presentation, *T*-cell biology, ruminant diseases

Immunology emerged as a scientific discipline in the end of nineteenth century, with the description of phagocytosis by Elie Metchnikoff and neutralizing antibodies by Emil Behring and Paul Ehrlich. For a long time, these major discoveries laid the bases of innate and acquired immunity, respectively (1). It was only in the early 1960's that observations by George Mackaness's team demonstrated for the first time that thymus-derived lymphoid cells (*T*-lymphocytes or *T*-cells) were able to transfer protection against infectious agents, allowing for the definition of cell-mediated immunity (CMI) (2). Since then, this arm of adaptive immunity, relying on the direct activity of antigen-specific *T*-cells rather than antibodies, has been associated to a variety of physiological processes including host response to infection, immune tolerance and resistance to cancer.

The biology of ruminant *T*-cells has been a topic of interest for immunologists for decades and the study of these cells has contributed to major observations on the immune system (3). Nevertheless, the scarcity of adapted tools, together with the dominance of rodent models, has limited the emergence of new discoveries on ruminant CMI and the expansion of a critical mass of experts in this field. To compensate for this shortcoming, this Research Topic reports experimental strategies to explore cellular adaptive immune responses in ruminants and provides new insight into these mechanisms in the context of physiological and pathological conditions.

Enzootic bovine leucosis (EBL) is a chronic contagious disease caused by the bovine leukemia virus (BLV), which compromises lymphocyte activities and leads to immune response dysfunction. Most of studies on the pathogenesis of EBL have been focused on the analysis of B and *T*-lymphocyte mechanisms (4). However, recent observations showing the role of neutrophils as regulators of both innate and adaptive immunity (5) raise questions about the role of these cells during EBL. Lv et al. explore the functional capacities of polymorphonuclear neutrophils isolated from dairy cows showing different BLV provirus loads. The authors demonstrate that BLV infection affects the immune function of neutrophils in a provirus load-dependent way, which could be associated to the higher incidence of infections, such as mastitis, in BLV-affected individuals.

Rainard et al. provide a broad review of reports on the adaptive immune response mechanisms in the mammary gland (MG) of ruminants, with an emphasis on cell-mediated immunity. Mammary gland is armed of physical and cellular defenses compatible with the production, storage, and secretion of milk, which differ from mechanisms found in other organs exposed to invaders. The immunological functions of mammary gland also varies greatly among mammals and this particularity limits extrapolations from one species to another (6). In this context, the authors compile the available knowledge, from pioneering to the most recent publications, to explore the specificities of cellular immunity in the MG of ruminants. They also discuss the major knowledge gaps concerning the mammary gland immunity that hamper the development of efficacious vaccines against intramammary infections.

Host immune response involves a myriad of complex processes difficult to mimic in laboratory conditions. For this reason, animal-based models are still necessary in different fields of immunology, including vaccine research and development. However, ethical and economic concerns involving animal experimentation have pushed the implementation of alternatives to *in vivo* observations and several *in vitro* immunogenicity models to explore the multiple functionally relevant parts of the immune response are now available (7). Davis et al. describe an *ex vivo* platform set out to study the primary and recall CD4^+^ and CD8^+^ *T*-cell responses to *Mycobacterium avium paratuberculosis* and *M. bovis* candidate vaccines. The authors also discuss the potential of cattle for the development of *ex vivo* models to investigate mechanisms evolved by pathogens to disturb immune response and cause persistent diseases, facilitating the access to knowledge necessary to the development of novel vaccines.

The description of dendritic cells represented a cornerstone for major discoveries in immunology. In the early 1990s, the emergence of protocols allowing the production monocytes-derived dendritic cells (moDC) paved the way for a better understanding of multiple processes of immune response including host response to infections and cancer, as well as immunotolerance (8). Although protocols for the production of bovine moDC based on the isolation of monocytes by magnetic sorting have been described (9, 10), the cost and the yield of this approach can represent a handicap. As an alternative, Cunha et al. propose cost and time-efficient protocols for the production of moDC and for the study of antigen presentation in bovine. These protocols might contribute to the setup of studies involving a large number of individuals, requiring a large number of cells or relying on the utilization of cryopreserved samples.

The immune checkpoint blockade has revolutionized the therapeutic landscape in oncology. Its success in cancer therapy implies that targeting similar pathways may prove beneficial in preventing and treating infectious diseases. Indeed, persistent infections are sustained by *T*-cell exhaustion, which is caused by the expression of immune checkpoint molecules that limit cell-mediated protective immunity (11). In this scenario, Souza et al. explore the role of the checkpoint molecules in the transition period and their association with the uterine health. Their observations might contribute to current efforts targeting the identification of resilient dairy cows and the development of innovative (immunological) non-antibiotic approaches covering the most critical period of the cow's life.

As a whole, this Research Topic proposes alternatives to some drawbacks involving the study of cell-mediated immunity in ruminants and highlights the better understanding of this arm of immune response as a fundamental step for the development of improved vaccines and therapies for these species.

## Author contributions

Research Topic conception by RPM. Research Topic editing and editorial writing by RPM and FNS. Both authors contributed to the article and approved the submitted version.
